# A Novel Recurrent Neural Network to Classify EEG Signals for Customers' Decision-Making Behavior Prediction in Brand Extension Scenario

**DOI:** 10.3389/fnhum.2021.610890

**Published:** 2021-03-08

**Authors:** Qingguo Ma, Manlin Wang, Linfeng Hu, Linanzi Zhang, Zhongling Hua

**Affiliations:** ^1^Institute of Neural Management Sciences, Zhejiang University of Technology, Hangzhou, China; ^2^School of Management, Zhejiang University, Hangzhou, China; ^3^School of Management, Zhejiang University of Technology, Hangzhou, China; ^4^School of Business Administration, Guizhou University of Finance and Economics, Guiyang, China; ^5^Shandong Apipi Education and Technology Co., LTD, Jining, China

**Keywords:** decision-making behavior, t-SNE, LSTM neural network, EEG, brand extension

## Abstract

It was meaningful to predict the customers' decision-making behavior in the field of market. However, due to individual differences and complex, non-linear natures of the electroencephalogram (EEG) signals, it was hard to classify the EEG signals and to predict customers' decisions by using traditional classification methods. To solve the aforementioned problems, a recurrent t-distributed stochastic neighbor embedding (t-SNE) neural network was proposed in current study to classify the EEG signals in the designed brand extension paradigm and to predict the participants' decisions (whether to accept the brand extension or not). The recurrent t-SNE neural network contained two steps. In the first step, t-SNE algorithm was performed to extract features from EEG signals. Second, a recurrent neural network with long short-term memory (LSTM) layer, fully connected layer, and SoftMax layer was established to train the features, classify the EEG signals, as well as predict the cognitive performance. The proposed network could give a good prediction with accuracy around 87%. Its superior in prediction accuracy as compared to a recurrent principal component analysis (PCA) network, a recurrent independent component correlation algorithm [independent component analysis (ICA)] network, a t-SNE support vector machine (SVM) network, a t-SNE back propagation (BP) neural network, a deep LSTM neural network, and a convolutional neural network were also demonstrated. Moreover, the performance of the proposed network with different activated channels were also investigated and compared. The results showed that the proposed network could make a relatively good prediction with only 16 channels. The proposed network would become a potentially useful tool to help a company in making marketing decisions and to help uncover the neural mechanisms behind individuals' decision-making behavior with low cost and high efficiency.

## Introduction

Neuroscience methods such as event-related potentials (ERPs) were widely used to investigate consumers' underlying thoughts, feelings, and intensions in marketing researches (Hsu, 2017). The core part of the ERP analysis was the analysis of the multichannel electroencephalogram (EEG) signals. However, the EEG signals recorded were featured with aperiodic, non-stationary, individual variance, and non-linear characteristics (Al Ghayab et al., 2019). Therefore, the development of classification methods to classify the multichannel EEG signals and to predict the decisions of the consumers were of great use.

In the literature, a lot of insightful researches on uncovering the behind neural mechanisms of some market scenarios could be found. For example, Ma et al. conducted ERP studies on brand extension (Ma et al., 2008, 2010; Wang et al., 2012); they found that some components of the EEG signal were related to the decision-making process in brand extension. Fu et al. (2019) investigated the impact of price deception on consumers' purchase intension by combining behavior and ERPs measures. They concluded an attenuated N2 and an increased late positive potential (LPP) under the truthful condition. Golnar-Nik et al. (2019) explored the impact of the advertisement on the consumers' shopping behaviors by using ERP methods. They performed feature extraction on the EEG spectral power, which had a very good prediction of decision-making incidence but with low preference classification accuracy. Jin et al. (2017) investigated the role of physical attractiveness played in online lending using ERP methods. They reported smaller N200 amplitude induced by attractive borrowers compared with the unattractive ones. They suggested the presence of the beauty premium phenomenon in online lending. As far as we are concerned, most of the current studies did not delve further into the EEG signals, and the relationship between the components of the signals and the customers' decision-making behavior was still unclear.

In recent decades, developing machine learning methods to directly classify EEG signals have attracted many scholars' attention. Lots of remarkable researches have been reported. These methods could be divided into three groups: convolutional neural network (CNN)-based classification methods, recurrent neural network (RNN)-based classification methods, and other methods. CNN-based classification methods were methods that proposed different CNN architectures to make classification by directly using EEG signals. For example, Sun, Lo, and Lo developed a novel approach based on 1D convolutional long short-term memory (LSTM) neural network to conduct the EEG-based user identification system (Sun et al., 2019). They validated the proposed method with a 109-subject public database and reported to have 99.58% accuracy. Jiao et al. proposed improved convolutional neural network (CNN) to classify mental load based on EEG data (Jiao et al., 2018). The authors compared their methods with the well-performed deep recurrent CNNs. They concluded that their methods achieved comparable or even better performance than the state-of-the-art ones but with less parameters. RNN-based methods were methods that developed different architectures of recurrent neural network and made classification. For example, Alhagry et al. (2017) adopted LSTM network to learn features from EEG signals and to classify these features into low/high arousal, valence, and liking. They reported an average accuracy of 85.65, 85.45, and 87.99% for different classes classification, respectively. Greaves (2015) investigated the effectiveness of multiple models on accurately classifying whether someone was viewing a 2D or 3D image. The authors compared the performance of a simple multilayer perceptron, a simple Elman recurrent neural network (RNN), and a time-dependent Elman RNN. Their results showed that the non-deep-learning approach outperformed the recurrent neural network models; moreover, a more complex RNN would have done better at classification. Meanwhile, the novel cascaded RNN architecture based on LSTM blocks to automatize sleep stages scoring using EEG signals was proposed by Michielli et al. (2019). They reported to have an overall 86.7% accuracy for five sleep stages classification. Other methods, such as Bode and Stahl (2014), used linear support vector machine classifier (LSVM) to predict errors from spatiotemporal patterns of ERPs (Bode and Stahl, 2014). Raghu and Sriraam (2018) developed an application called computerized automated detection of focal epileptic seizures (CADFES) to classify focal and non-focal EEG signals. Asadur Rahman et al. (2020) hybridized the principal component analysis (PCA) and t-statistics for feature extraction and compared the performance of four classifiers in emotion classification from multichannel EEG signal. Subasi and Gursoy (2010) proposed a signal processing and analysis framework for EEG, in which the signal were decomposed using discrete wavelet transform (DWT) and statistical features were extracted. They then adopted PCA, independent component analysis (ICA) to reduce the data dimension, and used SVM to classify the signals. La Foresta et al. (2009) adopted three methods (PCA, ICA, PCA-ICA) to extract the descriptive components from continuous coma—EEG to automatically detect the critical epochs. They concluded that the joint use of PCA-ICA had the best performance. Li et al. (2016) employed the parametric t-distributed stochastic neighbor embedding (t-SNE) to extract the non-linear features from MI-EEG and adopted SVM to classify the signals. They concluded an excellent classification performance. Neighborhood component analysis was also adopted in their research to assess the significant features. Accuracy of 96.1% using support vector machine with cubic kernel function was reported in their study. Although prior state-of-the-art studies have provided us with fruitful methods to directly classify EEG signals, for all we know, none of them had extended their methods in the field of marketing research. Whether, the existing methods could give a good prediction on customers' decision on brand extension by using the EEG signals obtained from the ERP paradigms still remained to be explored.

To address the aforementioned problems, we applied a brand extension ERP experiment as a market scenario and measured the EEG signals of the participants when they decided to accept a brand extension or not in current research. The proposed novel network architecture adopted t-SNE algorithm to automatically extract features from the EEG signals. Then, a recurrent neural network with LSTM layer, fully connected layer, and SoftMax layer was adopted to classify the EEG signals and to predict the success of brand extension. After that, we compared the proposed network with some commonly used networks, such as a recurrent PCA network, a recurrent ICA network, a t-SNE SVM network, a t-SNE BP neural network, a deep LSTM neural network, and a convolutional neural network to demonstrate its superiority in prediction accuracy. In addition, the performance of the network with different active channels was also investigated. Since the EEG signals were aperiodic, non-stationary, individual variance, and non-linear time series signals, we hypothesized that our method would outperform the other methods in accuracy by first applying t-SNE algorithm to extract features, which can retain the local neighborhood of dataset and reduce dimension, then adopting LSTM recurrent network to classify the time series features. Moreover, we hypothesized that the performance of proposed network in accuracy would increase as active channels increased; however, the predicted accuracy would converge with enough active channels.

The remaining parts of the paper were organized as follows: Materials and Methods introduced the experimental process, the data analysis method, and the proposed method. Results showed the experimental results and prediction results. Discussion and Conclusion presented the discussion and conclusion, respectively.

## Materials and Methods

The brand extension paradigm was adopted in this paper as a market scenario to collect the EEG signals and decision-making behaviors of the individuals, which were further used to build up the neural network for EEG signal classification and decision prediction (accept the brand extension or not).

### Experiment

#### Participants

Twenty-four participants aged 18–27 (12 female; M = 22.31, SD = 2.40) years old took part in the experiment with 40 yuan paid as volunteers. The recruited participants were all native Chinese speakers in good health and with normal or corrected-to-normal vision. Written consents were given to participants before the experiment; none of them reported a history of neurological disorder or mental disease. No alcohol and caffeinated drinks or smoking were allowed 1 day before the experiment. Among them, the experimental data of two participants were excluded, as there were insufficient trials to be superposed.

#### Experimental Procedure

The experiment instruction was offered for every participant to read first. Then, they were guided to sit comfortably in front of a display screen at 1 m away with a visual angle of 8.69° × 6.52° (15.2 × 11.4 cm, width × height) in a sound-attenuated, dark, electrically shielded room.

E-prime 2.0 software (Psychology Software Tools, Pittsburgh, PA, USA) was applied to perform the exponential stimulus. In the experiment, the stimuli were black words on white background. They were presented to each subject at the center of a computer-controlled video monitor.

**Figure 1:**
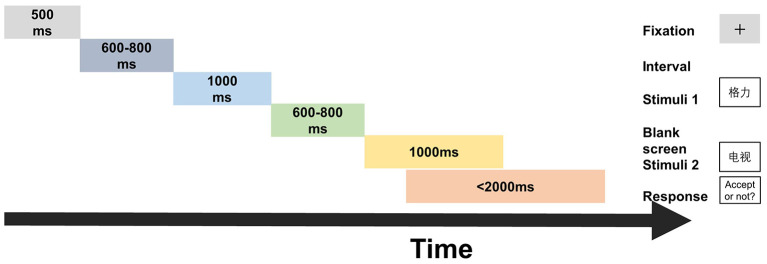
The brand extension paradigm: timeline for a brand extension task trail.

The brand extension paradigm is showed in Figure 1. In the experiment, each trial began with the sign of the fixation for 500 ms, following a random interval of 600–800 ms. Then, the stimulus 1, provided using the questionnaire software according to each participant's individual choices, was presented for 1,000 ms. A blank screen lasting from 600 to 800 ms at random was showed afterwards between stimulus 1 and stimulus 2. After that, stimulus 2 was presented for 1,000 ms. The participants were required to evaluate whether to accept the extension products in the second stimulus with brand name in the first stimulus and make a choice with keypad when stimulus 2 appeared. They were told to press the left button if they were willing to accept it, otherwise to press the right button in turn. The stimulus pairs (stimulus 1–stimulus 2) were presented at random. The whole experiment contained 216 trials. Twenty trials were designed for participants to practice to become familiar with the procedure; then, the remaining 196 trails were divided into four blocks each lasting about 7 min equally. There was a 5-min break after each block. Thus, the total experiment lasted about 45 min.

#### Experimental Stimuli

Two experimental stimuli, the brand name (stimulus 1) and product name (stimulus 2), were used in the experiment. All the stimuli were presented in Chinese, and the character of each stimuli was controlled in two or three. Stimulus 1 was all household brand names and was further divided into two groups: the familiar one and the unfamiliar one. Since each participants' familiarity with the specific household brands was different, we designed questionnaires for them to rate for 40 household brands (20 household appliance brands in real market and 20 artificial brands whose name resemble the real household appliance brands) based on their familiarity with each brand. For every participant, the seven most familiar brands and seven most unfamiliar brands would be picked up to compose the specific stimulus 1. We developed an in-lab Matlab code to present the questionnaires, which could output the brands to the experiment program automatically and quickly. Stimulus 2 was made up of 14 product names selected from 2 categories. Among them, seven were from beverage products (e.g., milk), while the remaining seven were from household appliance products (e.g., refrigerator). Thus, 14 brand names × 14 product names constituted the exponential stimuli. The list of the brands and products can be found in Table A1.

#### EEG Recording and Analysis

A Neuroscan Synamp2 Amplifier (Scan 4.3.1, Neurosoft Labs, Inc.) was applied to record EEG data continually (bandpass, 0.05–100 Hz; sampling rate, 1,000 Hz). The Ag/AgCl electrodes were placed at 64 scalp sites, and we took the left mastoid as reference with a cephalic (forehead) location as the ground. EEG recordings between −200 and 800 ms of S2 were extracted. The 200 ms prior to the S2 onset was corrected as baseline. The correction was done by subtracting the mean value of the EEG signals during the baseline period from each trial EEG signals. A regression-based algorithm (Semlitsch et al., 1986) implemented in the software Neuroscan 4.3 was then adopted to correct the electrooculogram artifacts with ocular movements. A digital filtering through a zero-phase shift using a low-pass filter at 30 Hz (24 dB/octave) followed. Trials in which peak-to-peak deflection exceeding ±80 μV were excluded from averaging. Trials in which there were more than 30 sweeps for each condition were retained. The final available trials per subject can be found in Table A2.

### Algorithm

In this section, we proposed an algorithm to predict customers' willing to accept the brand extension by directly using their EEG signals. Since EEG signal trials of different individuals are non-linear, non-stationary, and have low signal noise ratio, feature extraction algorithm was needed to extract features from the EEG signals for further classification. However, conventional feature extraction algorithm such as PCA and ICA were powerless to adequately extract relevant information from EEG signals (Amin et al., 2015). Therefore, t-SNE algorithm, which can retain the local neighborhood of dataset and reduce dimension (Li et al., 2016), was adopted here to extract the useful information from the EEG signals and kept in the original time sequence. Then, the time sequential features were inputted into the LSTM neural network, which has been proven as a commonly used neural network for classifying the time sequential signals.

#### Recurrent t-SNE Neural Network

**Figure 2:**
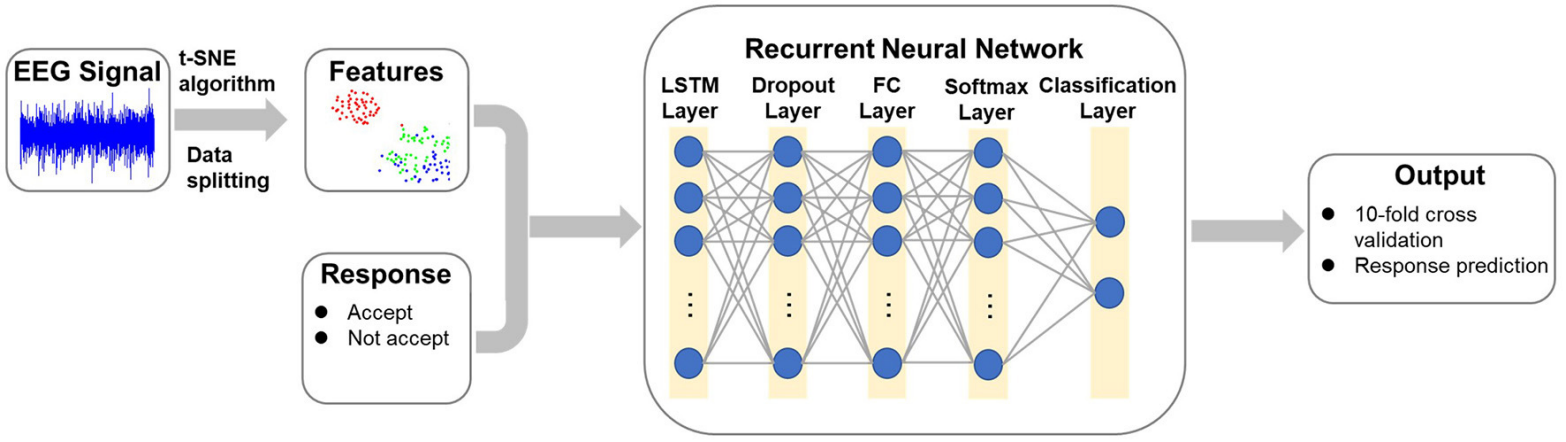
The architecture of the recurrent t-SNE neural network.

**Figure 3:**
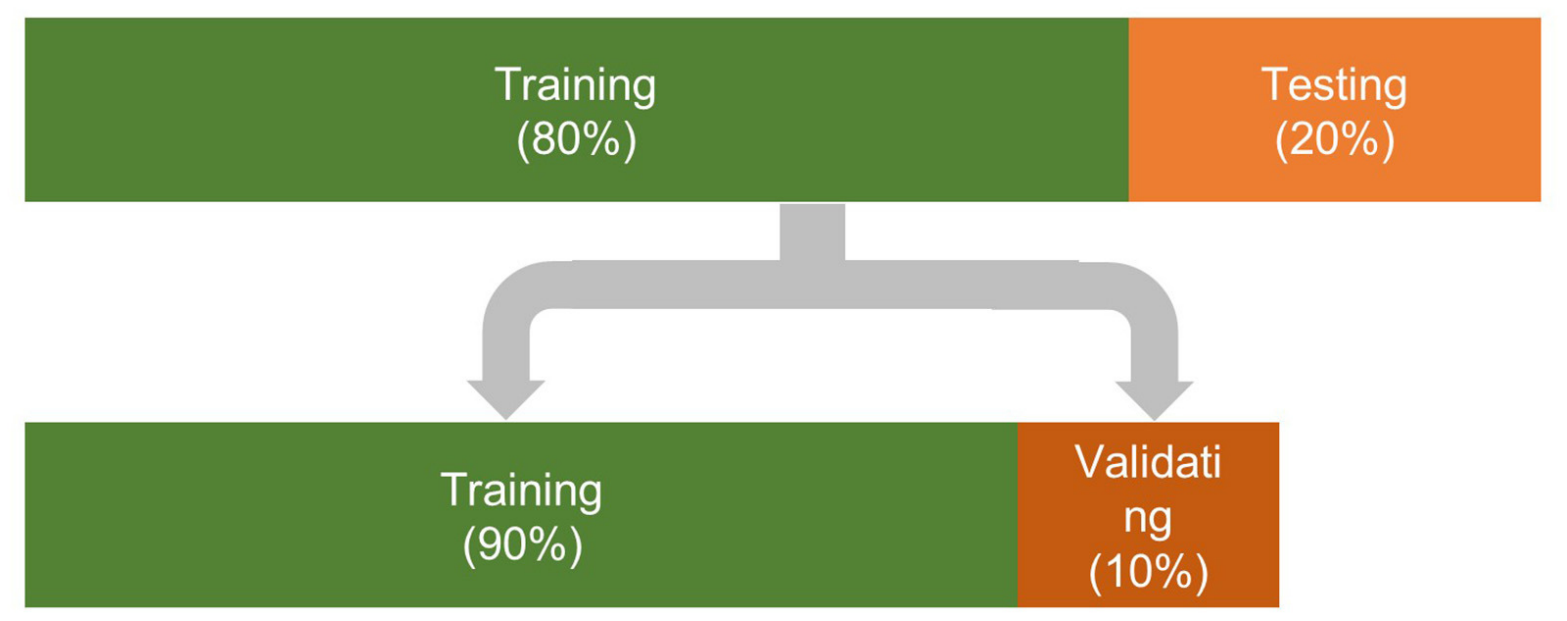
The allocation of dataset used for training and testing the proposed network.

A recurrent t-SNE neural network architecture was developed in this section to predict customers' willing to accept the brand extension product using the recorded EEG signals. The architecture of the proposed network is shown in Figure 2. The network included three parts: In the first part, the EEG signals and the corresponding responses were divided into three parts: the testing dataset, the training dataset, and the validating dataset. The allocation of dataset used for training, validating, and testing is shown in Figure 3. Then, a t-SNE algorithm was adopted to perform the feature extraction from the raw EEG signals and to reduce the size of the input data. In the second part, a recurrent neural network with LSTM layer, fully connected layer, and SoftMax layer was adopted to train the data and make the classification. Following the evaluation method applied in the previous study (Mao et al., 2014), the 10-fold leave-subject-out cross-validation strategy was adopted in this paper to evaluate the proposed network. In the last part, the test dataset was inputted into the trained network, and the predicted response was compared with the real response to test the predict accuracy of the proposed network.

#### t-SNE Algorithm

t-SNE algorithm (Maaten and Hinton, 2008) was developed by Maaten and Hinton to overcome the “crowding problem” and the difficulty in optimizing the cost function brought by the SNE algorithm. The SNE algorithm was proposed by Hinton and Roweis (2003) to convert the high-dimensional Euclidean distances between datapoints into conditional probabilities that represent similarities. Conditional probability *p*_*j*|*i*_ was employed to calculate the similarity of two datapoints *D*_*i*_ and *D*_*j*_. The expression of *p*_*j*|*i*_ was shown as follows (Hinton and Roweis, 2003):

(1) $$\begin{array}{l} p_{j|i}\;=\;\frac{\exp\left(-{\left\|D_{i}-D_{j}\right\|}^{2}/2\sigma_{i}^{2}\right)}{\underset{k\neq i}{\sum }\exp\left(-{\left\|D_{i}-D_{k}\right\|}^{2}/2\sigma_{i}^{2}\right)} \end{array}$$

where σ_*i*_ denoted the Gaussian variance centered on datapoint *D*_*i*_. To model the pairwise similarities, the value of *p*_*i*|*i*_ was set as 0. The conditional probability *q*_*j*|*i*_ of the low-dimensional counterparts *d*_*i*_ and *d*_*j*_ were denoted as follows:

(2) $$\begin{array}{l} q_{j|i}\;=\;\frac{\exp\left(-{\left\|d_{i}-d_{j}\right\|}^{2}\right)}{\underset{k\neq i}{\sum }\exp\left(-{\left\|d_{i}-d_{k}\right\|}^{2}\right)} \end{array}$$

Similarly, *q*_*i*|*i*_ was set as 0. Ideally, the conditional probabilities *p*_*j*|*i*_ and *q*_*j*|*i*_ would be equal if the mapping was correct. Therefore, the aim of the SNE algorithm was to find a low-dimensional data representation that minimizes the mismatch between *p*_*j*|*i*_ and *q*_*j*|*i*_. The algorithm adopted Kullback–Leibler divergence to measure the mismatch between *p*_*j*|*i*_ and *q*_*j*|*i*_ (Hinton and Roweis, 2003). The cost function C was given by:

(3) $$\begin{array}{l} C\;=\;\underset{i}{\sum }KL\left(P_{i}\|Q_{i}\right)=\underset{i}{\sum }\underset{j}{\sum }p_{j|i}\log\frac{p_{j|i}}{q_{j|i}} \end{array}$$

where *P*_*i*_ denoted the conditional probability distribution over all other datapoints given by datapoint *D*_*i*_, and *Q*_*i*_ denoted the conditional probability distribution over all other map points given by map point *d*_*i*_.

t-SNE algorithm improved SNE algorithm by replacing the Gaussian distribution with a heavy-tailed student t-distribution in the low-dimensional space and adopted a symmetric version of the SNE cost function, yielding *p*_*j*|*i*_ = *p*_*i*|*j*_ and *q*_*j*|*i*_ = *q*_*i*|*j*_ (Maaten and Hinton, 2008; Li et al., 2016). The improvement was found to be useful in solving the “crowding problem” and the difficult cost function optimization problem. The joint probabilities *q*_*j*|*i*_ in t-SNE algorithm was given by:

(4) $$\begin{array}{l} q_{j|i}\;=\;\frac{{\left(1+{\left\|d_{i}-d_{j}\right\|}^{2}\right)}^{-1}}{\underset{k\neq i}{\sum }{\left(1+{\left\|d_{k}-d_{i}\right\|}^{2}\right)}^{-1}} \end{array}$$

The gradient of the Kullback-Leibler divergence between *P*_*i*_ and *Q*_*i*_ was given by:

(5) $$\begin{array}{l} \frac{\delta C}{\delta d_{i}}\;=\;4\underset{j}{\sum }\left(p_{j|i}-q_{j|i}\right)\left(d_{i}-d_{j}\right){\left(1+{\left\|d_{i}-d_{j}\right\|}^{2}\right)}^{-1} \end{array}$$

By employing the t-SNE algorithm, the size of the raw EEG signals could be reduced, and the obtained data could be used as the features of the EEG signals. The features combined with the corresponding response were then further divided into three parts (Figure 3) to train, validate, and test the recurrent neural network.

#### Recurrent Neural Networks

**Figure 4:**
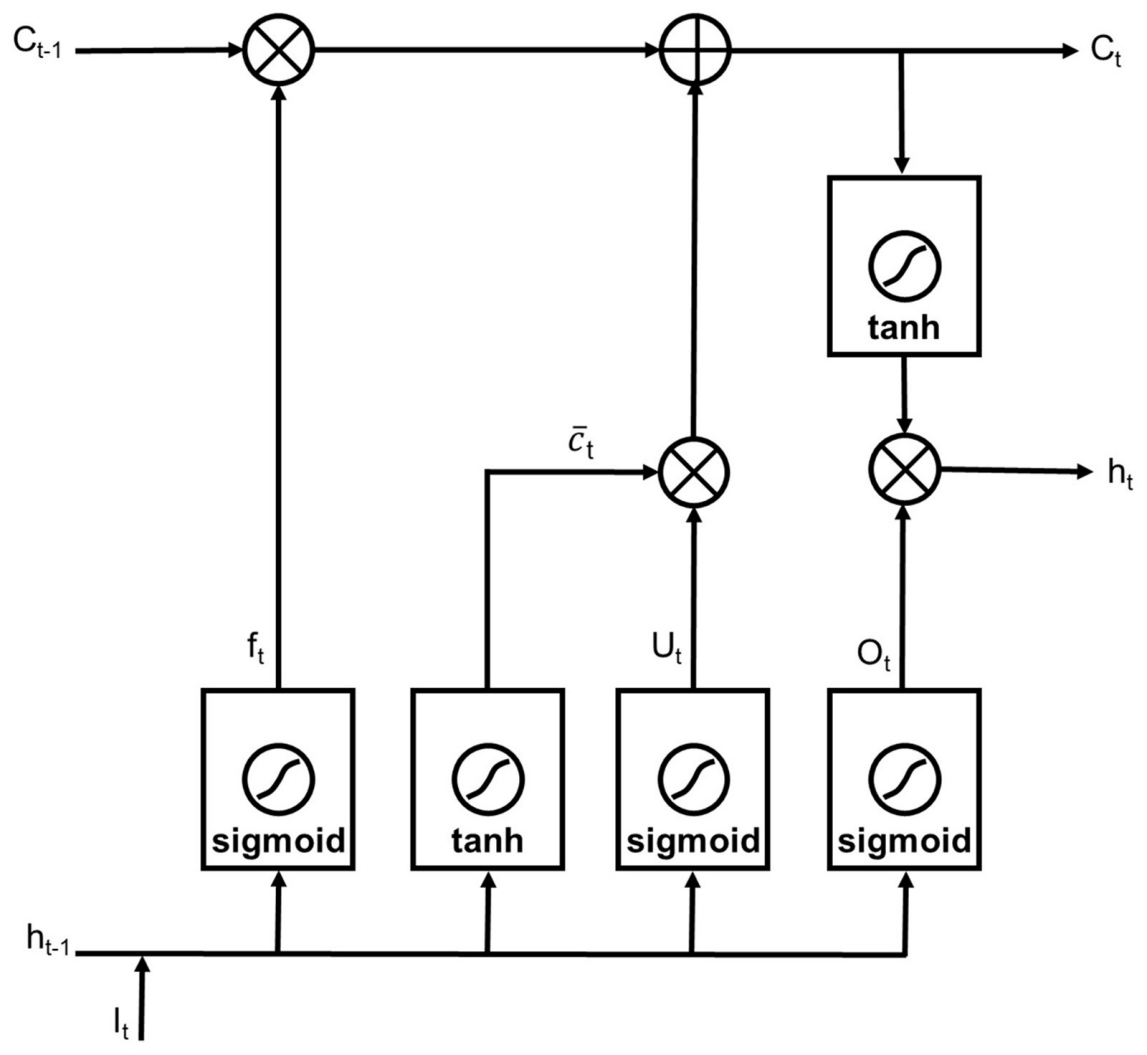
The architecture of a LSTM layer.

The adopted RNN was composed of a LSTM layer, a dropout layer, a fully connected (FC) layer, a SoftMax layer and a two-category classification layer (Figure 2). The LSTM network was one of the recurrent neural network architecture introduced by Hochreiter and Schmidhuber to better capture long-term dependencies and to address the problem of vanishing gradient of the standard RNN (Hochreiter and Schmidhuber, 1997). The LSTM layer consisted of five components: the memory cell *C*_*t*_, the candidate value ${\bar{c}}_{t}$, and three gates defined as forget gate *f*_*t*_, update gate *U*_*t*_, and output gate *O*_*t*_. The architecture of a LSTM layer is plotted in Figure 4.

The memory cell was the cell to be updated at each timestep, while the candidate value was the value to replace the memory cell at each timestep. The function of the forget gate was to decide which information to throw away from the cell state by using a sigmoid layer. The expression of the forget gate was given by:

(6) $$\begin{array}{l} f_{t}=\sigma \left(W_{f}\cdot \left[h_{t-1},I_{t}\right]+b_{f}\right) \end{array}$$

The function of the update gate was to decide whether to replace the memory cell with the candidate value or not. The expression of the update gate was given by:

(7) $$\begin{array}{l} C_{t}\;=\;f_{t}*C_{t-1}+U_{t}*{\bar{c}}_{t} \end{array}$$

(8) $$\begin{array}{l} {\bar{c}}_{t}=\tanh\left(W_{c}\cdot \left[h_{t-1},I_{t}\right]+b_{c}\right) \end{array}$$

(9) $$\begin{array}{l} U_{t}=\sigma \left(W_{U}\cdot \left[h_{t-1},I_{t}\right]+b_{U}\right) \end{array}$$

The output gate decided where the activation at the current timestep was generated. The expression of the output gate was given by:

(10) $$\begin{array}{l} O_{t}=\sigma \left(W_{o}\cdot \left[h_{t-1},I_{t}\right]+b_{o}\right) \end{array}$$

(11) $$\begin{array}{l} h_{t}=O_{t}*\tanh\left(C_{t}\right) \end{array}$$

where σ denoted a sigmoid activation function, *I*_*t*_ was the input vector, and tanh denoted hyperbolic tangent activation function. *W*_*f*_, *W*_*c*_, *W*_*U*_, and *W*_*o*_ were the weight matrix, and *b*_*f*_, *b*_*c*_, *b*_*U*_, and *b*_*o*_ are the bias terms. The notation * denoted the Hadamard product, and *h*_*t*_ was the past hidden state.

In the employed recurrent neural network, after passing through the LSTM layer, a dropout layer developed by Srivastava et al. (2014) to avoid the overfitting of the neural network was adopted in current research to avoid the overfitting of the LSTM neural network. Its principal idea was to randomly drop units from the neural network during training to prevent units from coadapting too much. Then, a fully connected layer was used for further classification. A SoftMax layer and a two-category classification were used at the end for response prediction.

#### Training

To validate the proposed recurrent t-SNE neural network, a commonly used k-fold (k = 10) cross-validation was adopted. In each validation, 20% of the data were reserved for testing, while the remaining data were used for training/validating. The training and validating data were selected randomly. Their ratio was 9:1. They were shuffled and randomly selected into batches for each iteration of training. Each batch contained 100 sets of 1,000 × *N*_chan_ EEG signals, depending on the activated channels. The training of the networks was stopped when reaching 20 epochs. The sgdm algorithm was chosen to optimize the neural network. The dropout rate and the learning rate were set to 0.2 and 0.001, respectively.

## Results

In this part, the performance of the proposed recurrent t-SNE neural network and comparisons with the performance of other prediction methods were shown. The comparison prediction methods could be divided into three groups. For the first group, three commonly used feature extraction methods were applied to substitute the feature extraction method in proposed recurrent t-SNE neural network. Therefore, there were a recurrent PCA neural network, a recurrent ICA neural network, and a deep LSTM neural network in the first group of methods. For the second group of methods, we used other classification methods to replace the LSTM neural network. Therefore, we had a t-SNE SVM network and a t-SNE BP neural network belonging to the second group of methods. For the third group of methods, the widely used convolutional neural network was trained with the EEG time–frequency diagrams, and its performance was compared with the proposed network. Moreover, the performance of the proposed network trained with different number of channels was also investigated, and the predicted results were also compared.

### Prediction Results

The setup parameters for the proposed recurrent t-SNE neural network are shown in Table 1. The t-SNE algorithm was adopted to extract features from the EEG signal. The sequence-to-label data architecture was inputted into the LSTM layer, and the layer was set to have 80 hidden units. The value of the dropout rate for the dropout layer was set to be 0.2, and the fully connected layer was set to have 2 units. The training process was conducted on a laptop with a 2.6-GHz Intel Core (TM) i7-6700HQ CPU, 8 GB of memory RAM and 64-bit version of Windows. The training platform was the MATLAB 2019a version.

**Table 1:** The setup parameters for different neural networks.

**Order**	**Method**	**Algorithm and layer type**	**Properties**
1	a recurrent t-SNE neural network	t-SNE algorithm	Extract features
2		LSTM layer	80 hidden units, sequence-to-label architecture
3		Dropout layer	Dropout rate 0.2
4		Fully connected layer	2 units
5		SoftMax layer	Softmax activation function
6		Classification layer	2 classes
1	t-SNE SVM method	t-SNE algorithm	Extract features
2		SVM classification method	Radical basis function
1	t-SNE BP neural network	t-SNE algorithm	Extract features
2		Fully connected layer	20 hidden units
3		Fully connected layer	10 hidden units
4		Output layer	1 unit
5		SoftMax layer	Softmax activation function
1	A recurrent PCA neural network	PCA algorithm	Extract features
2		LSTM layer	80 hidden units, sequence-to-label architecture
3		Dropout layer	Dropout rate 0.2
4		Fully connected layer	2 units
5		Softmax layer	Softmax activation function
6		Classification layer	2 classes
1	A recurrent ICA neural network	ICA algorithm	Extract features
2		LSTM layer	80 hidden units, sequence-to-label architecture
3		Dropout layer	Dropout rate 0.2
4		Fully connected layer	2 units
5		SoftMax layer	SoftMax activation function
6		Classification layer	2 classes
1	A deep LSTM neural network	LSTM layer	Extract features
2		LSTM layer	80 hidden units, sequence-to-label architecture
3		Dropout layer	Dropout rate 0.2
4		Fully connected layer	2 units
5		SoftMax layer	SoftMax activation function
6		Classification layer	2 classes
1	A convolutional neural network	Image input layer	Input images
2		2D Convolution layer	20 filters of size [5 5]
3		Batch normalize layer	
4		Relu layer	Relu activation function
5		Fully connected layer	2 units
6		Softmax layer	Softmax activation function
7		Classification layer	2 classes

**Figure 5:**
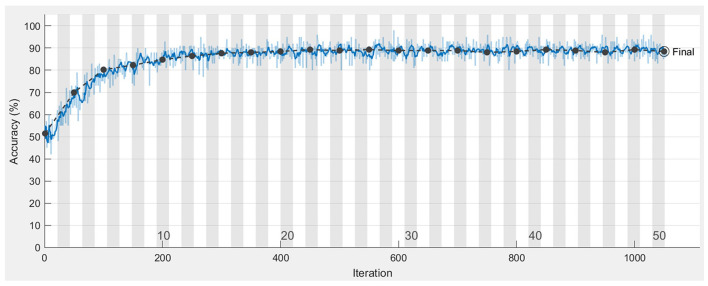
Training and validation accuracy over iterations for the proposed recurrent t-SNE neural network.

The robustness and prediction accuracy of the proposed recurrent t-SNE neural were tested by 10-fold cross-validation. The training and validation process for one of the validations is shown in Figure 5. The blue and black lines represented the training and validation accuracy over the iterations, respectively. Observed from Figure 5, we found that the training and validation accuracy increased and then converged to a certain accuracy as the training iteration increased. No overfitting occurred during the training, and the accuracy of this training finally converged to 88.48%. The average prediction accuracy for the 10-fold validation was 87.37%, and the standard deviation of the prediction accuracy was 1.78%.

### Compare With Other Prediction Methods

Three groups of methods were employed to train the EEG signal of brand extension experiment and predicted the success of the brand extension. The architectures of the methods were introduced, and the predicted accuracy was summarized and compared.

#### Group 1 Methods

Besides t-SNE feature extraction method and other methods, such as PCA, ICA, and LSTM, feature extraction methods could also be used to extract features from the EEG signals. In the current research, t-SNE method was replaced by PCA, ICA, and LSTM method, respectively, to form a recurrent PCA neural network, a recurrent ICA neural network, and a deep LSTM neural network. The setup parameters for these networks are shown in Table 1. PCA is a kind of principal component analysis method, which has been widely used in feature extraction and data compression (Praneeth et al., 2017; Salo et al., 2019). The principal method was using orthogonal transformations to convert the data into a set of linearly uncorrelated components without losing much information. In the study, the EEG signal of 1,000 datapoints was converted into five principal components by using the PCA method. ICA method was a blind source separation method to recover independent source signals from the raw signals (Tong et al., 2014; Jiang et al., 2019). The method assumed that the signals were non-Gaussian signals and were statistically independent from each other. In this premise, ICA could separate the signal through a dealiasing system. In this paper, the ICA method was adopted to decompose the EEG signals into five independent components. A deep LSTM neural network was developed by using a LSTM layer and a dropout layer to extract the features serving as the input of the recurrent neural network. The added LSTM layer had 120 hidden units and was set to be sequence to sequence architecture. The dropout rate of the following dropout layer was set to be 0.2. Ten-fold cross-validation was also performed on these networks to see the average and standard deviation of the validation accuracy. The average validation accuracy for the recurrent PCA neural network, recurrent ICA neural network, and deep LSTM neural network were 58.02, 59.18, and 58.35%, respectively. The standard deviation for these networks were 4.06, 2.33, and 2.63%, respectively. None of these networks had a better performance in predicting the participants' choices.

#### Group 2 Methods

Group 2 methods were trying to replace the recurrent neural network with other classification methods, such as support vector machine and a simple back-propagate neural network. In the research, we probed into the performance of the t-SNE SVM method and t-SNE BP neural network by using 10-fold cross-validation. The architectures of the t-SNE SVM method and t-SNE neural network are shown in Table 1. The t-SNE SVM method adopted t-SNE algorithm to decompose the EEG signals into five-dimensional data, and then, the SVM method with radical basis function kernel was used to classify the low-dimensional data. The t-SNE neural network also employed t-SNE algorithm to decompose the EEG signals into five-dimensional data and used back propagation neural network to classify the low dimensional data. The back propagation neural network contained four layers: 2 fully connected layers with 20 and 10 hidden units, 1 output layer with 1 unit, and 1 SoftMax layer. The average validation accuracy for the t-SNE SVM method and the t-SNE BP neural network were 53.29 and 40.69%, respectively. The standard deviations for the two networks were 3.84 and 3.10%, respectively. None of the two networks had a better performance in predicting the success of the brand extension. Moreover, the t-SNE neural network even presented a prediction <50%, which was even worse than a random guess.

#### Group 3 Method

For Group 3 method, a two-dimensional convolutional neural network was employed to train the data and make the prediction. The setup parameters for the convolutional neural network are shown in Table 1. The EEG signals were transformed to time–frequency diagrams by using continuous wavelet transformation. Then, the diagrams were inputted into the convolutional neural network for training, validating, and testing. The convolutional neural network contains seven layers. The first layer was an image input layer to input time–frequency diagrams into the network. The second layer was a 2D convolution layer, which had 20 filters of size [5 5], followed by a batch normalize layer that normalized each input channel across a mini batch. A Relu layer was used after that to perform a threshold operation to each element of the input, where any value <0 was set to 0. Finally, 2 units fully connected layers, SoftMax layer, and two-class classification layer were used to classify the data and make prediction. The average validation accuracy for the convolutional neural network was 60.19%, and the standard deviation for the convolutional neural network was 1.35%.

**Figure 6:**
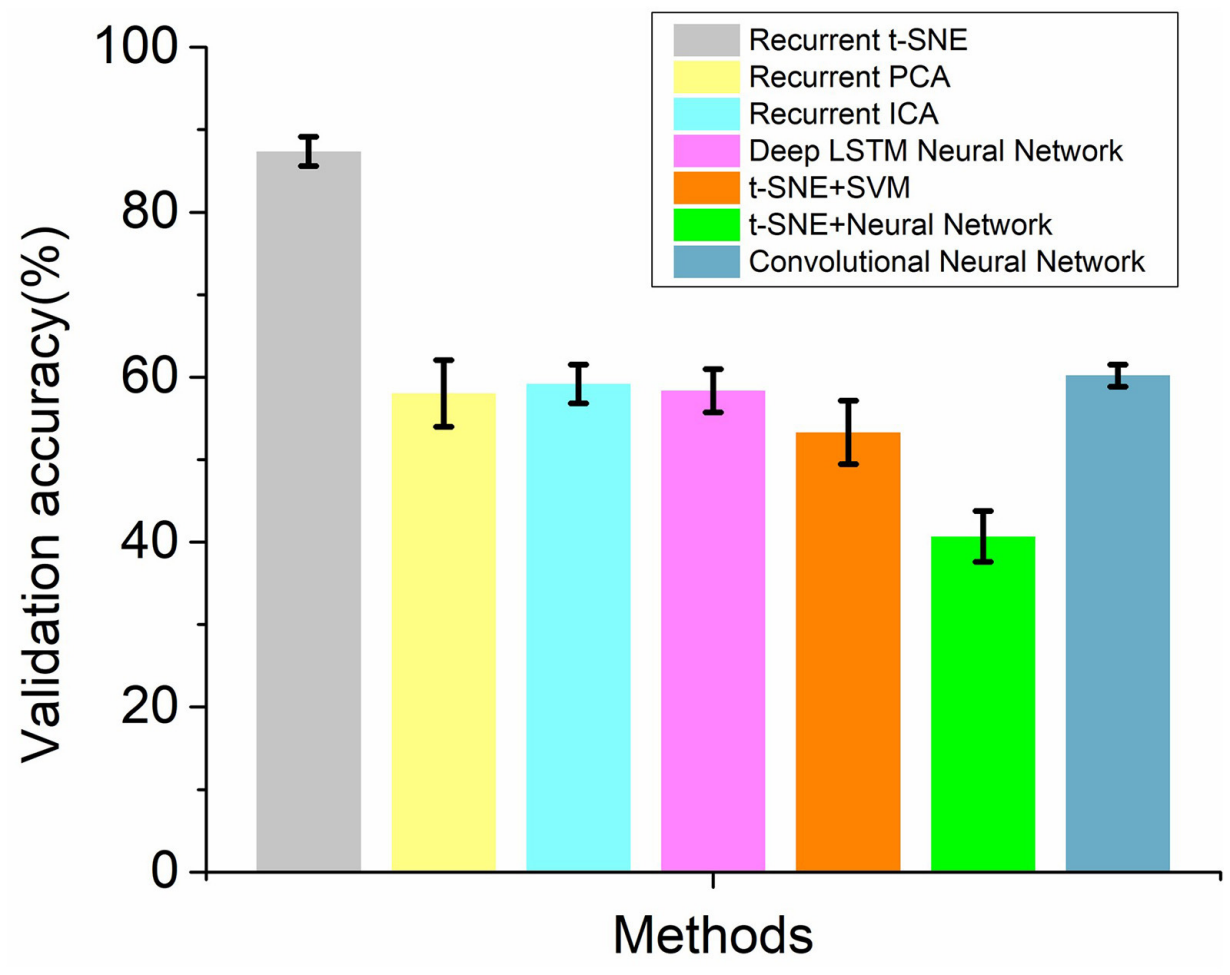
The comparison of validation accuracies of the recurrent t-SNE method and 3 groups of other methods.

The comparison of the recurrent t-SNE method and three groups of other methods is shown in Figure 6. It could be observed from the results that the average validation accuracy for recurrent t-SNE neural network was much higher than the validation accuracies of other methods. The standard deviation for the validation accuracy for all the networks were all smaller than 5%, which indicated that all the methods were robust. The proposed recurrent t-SNE neural network was better than other compared methods in predicting customers' decision-making behaviors.

**Figure 7:**
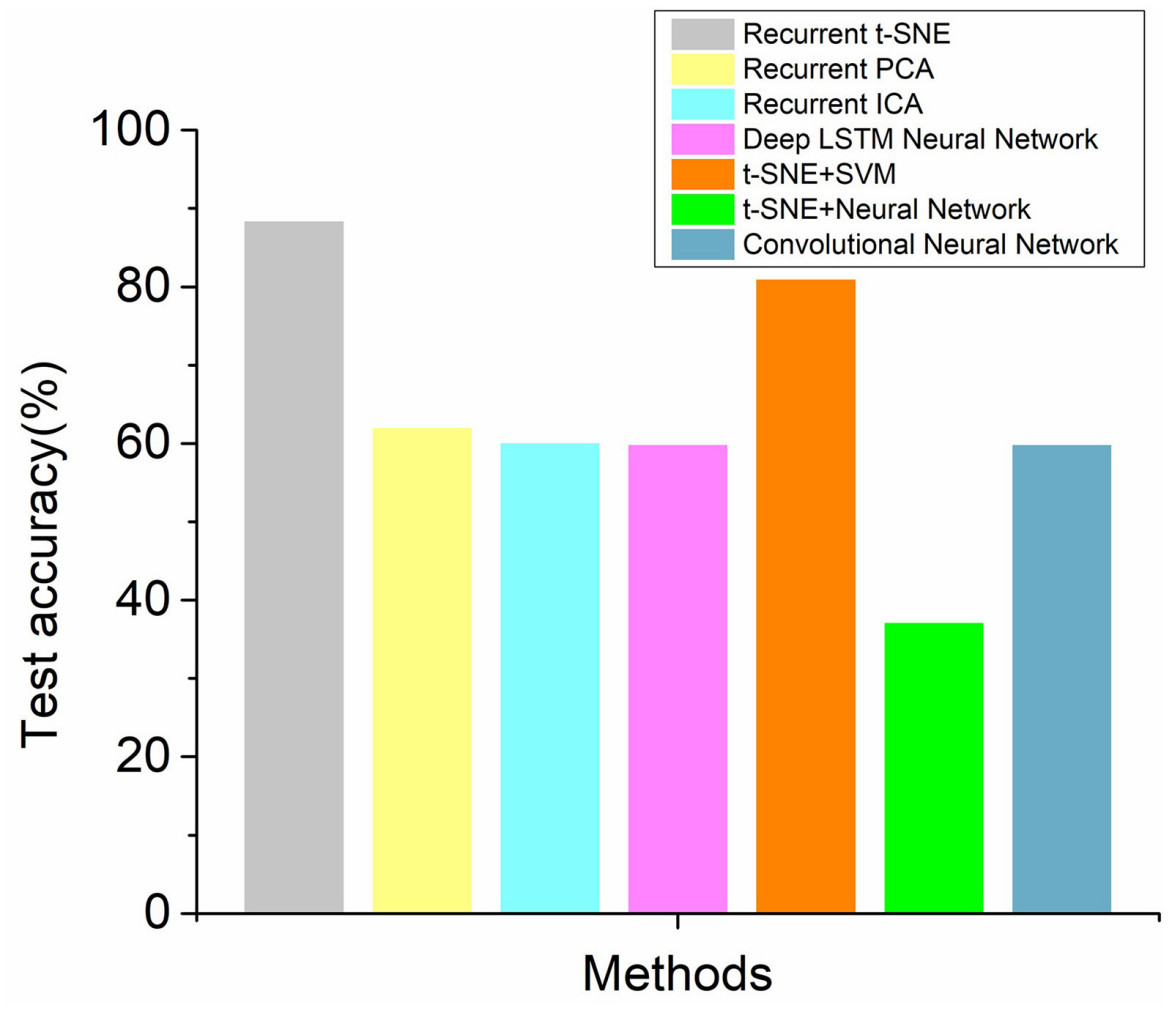
The comparison of test accuracies of the recurrent t-SNE method and 3 groups of other methods.

The test accuracies for the recurrent t-SNE neural network and other methods are shown in Figure 7 by evaluating different methods with test dataset. It could also be observed from Figure 7 that the proposed recurrent t-SNE neural network outperformed other methods in testing accuracy. The testing accuracies of most of the methods were close to the validation accuracies, which indicated the generalization ability of the methods. However, the testing accuracy of the t-SNE SVM method was much higher than the validation accuracy, which indicated that the method was hard to be applied to new dataset.

### Prediction Results With Different Channel EEG Signal

**Figure 8:**
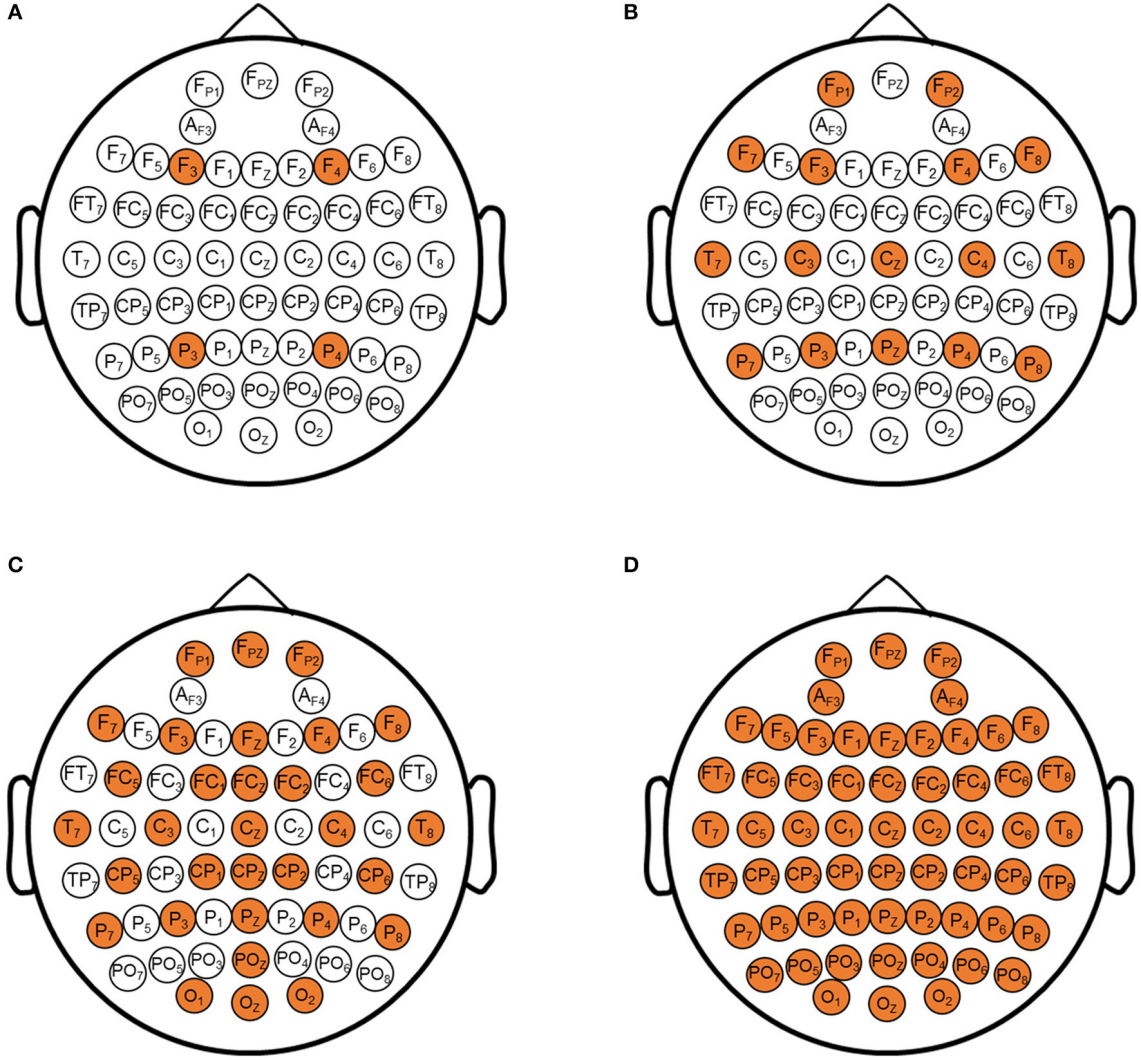
The selected electrode positions on scalp for active channels. **(A)** 4 electrode positions; **(B)** 16 electrodes positions; **(C)** 32 electrodes positions; **(D)** 64 electrodes positions.

**Figure 9:**
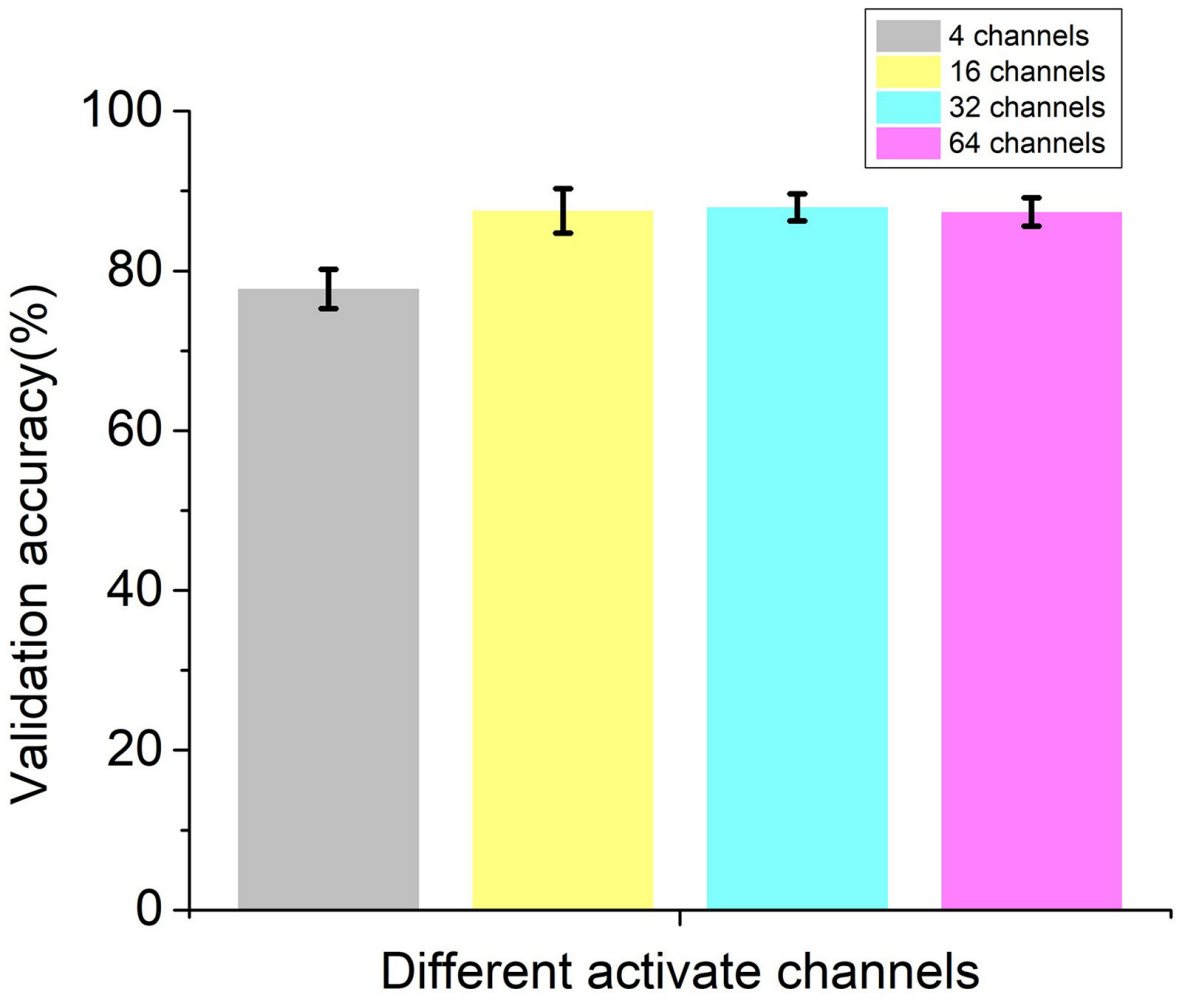
Validation accuracies of the recurrent t-SNE neural network for different active channels.

The performance of recurrent t-SNE neural network with different activated channels was also evaluated in this section. The aim of the comparison was to see if the proposed network could give a good prediction with less electrode positions, which meant to lower the experimental cost. The selected electrode positions on the scalp for active channels are depicted in Figure 8, considering the positions in the literature (Sun et al., 2019) and our test equipment. Figures 8A–D show the position of 4, 16, 32, 60 active channels, respectively. The predicted results with different active channels are shown in Figure 9.

**Figure 10:**
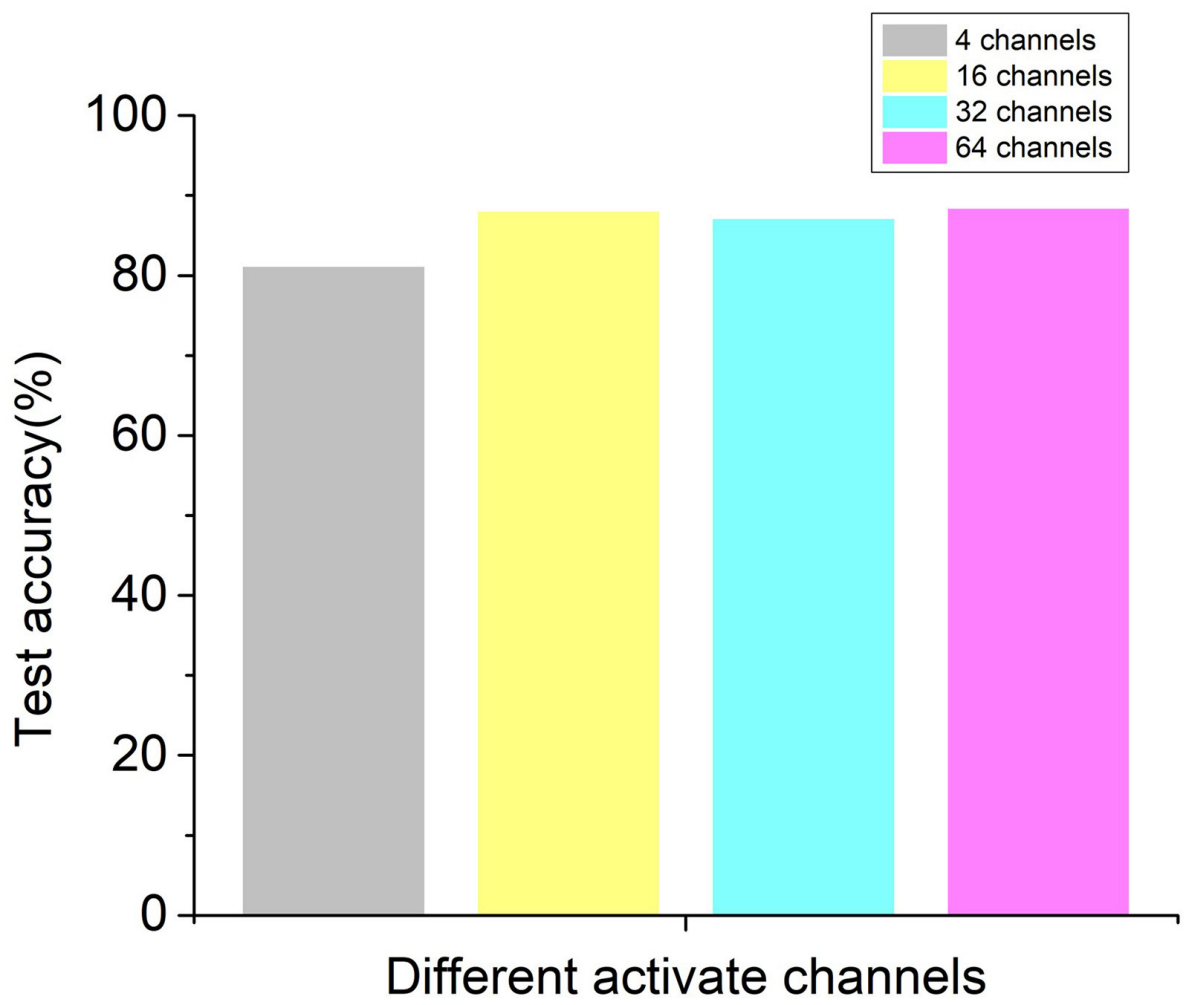
Test accuracies of the recurrent t-SNE neural network for different active channels.

As shown in Figure 9, the average validation accuracy for the proposed recurrent t-SNE neural network was 77.74, 87.49, 87.94, and 87.37% for 4, 16, 32, and 60 active channels, respectively. The standard deviation for 4, 16, 32, and 60 active channels was 2.47, 2.78, 1.71, and 1.78%, respectively. The proposed neural network had a poor performance with only four active channels and had a good performance in all other three conditions. However, it could be observed from Figure 9 that with 16 active channels or more, the validation accuracies were close to each other and tended to converge. The results indicated that 16-channel EEG signals were enough to make a good prediction of consumers' decisions and save training time and cost of the experiments. Figure 10 shows the test accuracies of the recurrent t-SNE neural network for different active channels. The same trend could be observed. The test accuracies with different active channels were close to their validation accuracies, which indicated good generalization of the proposed recurrent t-SNE neural network.

## Discussion

In the current research, a recurrent t-SNE neural network architecture was proposed to classify the EEG signals for consumer's decision-making behavior prediction in a brand extension scenario. With the average validation accuracy of 87.37% (standard deviation = 1.78%) in a 10-fold cross-validation and test accuracy of 88.32%, the proposed architecture could be declared as an effective method to automatically bridge the EEG signal modulated by the participants' response (accept or not) in the brand extension process.

The proposed network was also compared with three groups of other methods. Group 1 methods replaced t-SNE method with other feature extraction methods. Group 2 methods replaced the recurrent neural network with SVM and back propagation neural network. Group 3 employed the widely used convolutional neural network. The proposed network outperformed these three group of methods with more than 20% prediction accuracy. The comparison results indicated that the t-SNE feature extraction method combined with the proposed recurrent neural network was better than other neural network with other combinations. Scholars working on brain computer interface also proposed many other deep learning architectures to classify the EEG data in different experiments recently, for instance, Deep Belief Net (Ahmed et al., 2013), 1D-convolutional long short-term memory neural network (Sun et al., 2019), Deep Riemannian Model (Hajinoroozi et al., 2017), etc. Most of the studies were the improved architectures of the convolutional neural network, which took huge amount of training time. Since the research could not exhaust all the existing networks and the networks did not provide all the layer information and hyperparameters, we did not investigate them in the current study.

Nevertheless, compared to the Group 3 method, a simple convolutional neural network, our method was superior in accuracy and also required fewer training times (several minutes vs. several hours). Since we did not exhaust all the existing methods, the proposed neural network could only be claimed to be a good method to predict the consumers' choice in brand extension and maybe the best. The above comparison results supported our hypothesis.

The performance of the proposed neural network with different activate channels (4, 16, 32, 60) was also investigated in this paper. The results indicated that with 16 active channels, the proposed neural network could give a good prediction and save the training and experimental cost. Although the results showed that the performance with 32 active channels can have 0.5% improvement in validation accuracy, it was not worth to invest much more training and experimental cost for that negligible improvement in most cases. The results were in accord with our hypothesis.

**Figure 11:**
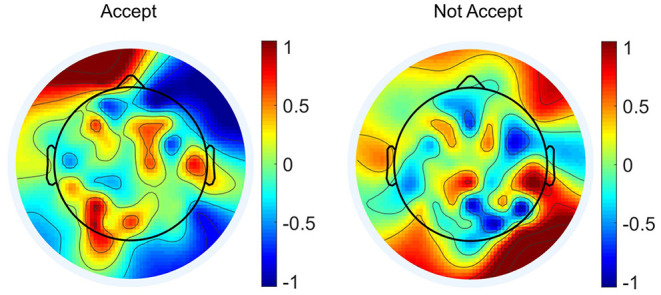
Normalized feature values mapped onto the scalp for accept and not accept conditions.

Moreover, the proposed network was helpful to find out the possible active brain zones when participants were making different decisions. Figure 11 shows the normalized feature values mapped on the scalp for accept and not accept conditions. The feature values were averaged across the five datapoints after performing t-SNE algorithm to the EEG signal. Then, the features values were normalized by the maximum feature value of all the channels. It could be clearly observed from Figure 11 that the active brain zones and the values were different for two different conditions. Therefore, the neural mechanisms behind accepting or not the brand extension could be carefully studied by relating the functions of different brain zones to the choices, which could be a valuable basis for future work.

The proposed recurrent t-SNE neural network was a novel method in neuroscience, which had the benefit of fast and accurately in classifying the EEG signals and predicting customers' decision in brand extension scenario. The network had potential in predicting consumers' decision in real market circumstances with lower cost and higher efficiency and in providing new perspectives for future research.

## Conclusion

In the study, a recurrent t-SNE neural network was proposed to automatically extract features from the EEG signals, classify the EEG signals, and predict consumers' choice in a brand extension scenario. Several conclusions were summarized as follows:

1. By combining the t-SNE algorithm and the recurrent neural network, the proposed neural network could well predict the response of the subjects by directly using the EEG signals. The average validation accuracy was 87.37% (standard deviation = 1.78%) in a 10-fold cross-validation, and test accuracy was 88.32%.
2. Compared with many other methods [a recurrent PCA network, a recurrent independent component correlation algorithm (ICA) network, a t-SNE SVM network, a t-SNE BP neural network, a deep LSTM neural network, and a convolutional neural network], the proposed recurrent t-SNE neural network demonstrated its superior in prediction accuracy.
3. By comparing the performance of the proposed neural network with different channels, we found that 16 active channels were the best choice in most instances and that the prediction accuracy and the experimental and training cost could achieve balance to a certain degree.

With the proposed neural network, the active brain zones under different conditions could be found out and help to predict the consumers' behavior with low cost. Moreover, the method could be a potential tool to help the company in making critical marketing decisions.

## Data Availability Statement

The data analyzed in this study is subject to the following licenses/restrictions: Data can be sent to the readers if they request to access the data after it is published. Requests to access these datasets should be directed to QM, maqingguo3669@zju.edu.cn.

## Ethics Statement

The studies involving human participants were reviewed and approved by the Neuromanagement Laboratory Ethics Committee at Zhejiang University. The participants provided written informed consent to participate in this study.

## Author Contributions

QM designed the experiment. MW and LH finished the experiment and data collection. MW and LZ finished the neural model coding. All the authors wrote the draft and revised the manuscript.

## Conflict of Interest

ZH was employed by the company Shandong Apipi Education and Technology Co., LTD. The remaining authors declare that the research was conducted in the absence of any commercial or financial relationships that could be construed as a potential conflict of interest.

## Appendix

**Table A1:** List of brands and products.

**REAL BRANDS**				
Midea	Philips	Gree	Panasonic	Changhong
Konka	Skyworth	Supor	Hisense	LittleSwan
Joyoung	Povos	Galanz	Flyco	Aux
Donlim	Sacon	Vatti	Xiaoya	Braun
**ARITIFICAL BRANDS**				
Aimeite	Whirlpool	Jiahe	Wanhe	Huaxin
Oushilan	Leda	Xiongpu	Molong	Huaxunshi
Delong	Pengshangde	Dika	Chuangyuexin	Shihailun
Huangwei	Kaiyou	Gangde	Aodingmei	Gaoxinke
**PRODUCTS**				
Milk tea	Soda water	Fruit juice	Green tea	Milk
Red tea	Soy milk	TV	Color TV	Refrigerator
Electric fan	Fan	Air conditioner	Freezer	

**Table A2:** Trial number of each subject under each condition.

**Subject**	**High-familiarity and high-conflict product category**	**High-familiarity and low-conflict product category**	**Low-familiarity and high-conflict product category**	**Low-familiarity and low-conflict product category**
1	40	38	38	40
3	30	34	31	31
4	37	37	37	39
5	38	40	39	39
6	40	37	40	38
7	32	36	30	33
8	32	31	34	36
9	40	40	40	39
11	47	45	48	46
12	50	45	48	46
13	34	32	31	36
14	44	43	47	47
15	49	49	49	47
16	47	45	43	46
17	39	39	38	37
18	47	49	49	49
19	40	46	43	42
20	48	49	49	49
21	48	48	44	48
22	49	49	48	47
23	49	47	49	47
24	44	47	47	41
